# An updated synopsis of *Tanaecium* (Bignonieae, Bignoniaceae)

**DOI:** 10.3897/phytokeys.132.37538

**Published:** 2019-09-26

**Authors:** Annelise Frazão, Lúcia G. Lohmann

**Affiliations:** 1 Universidade de São Paulo, Instituto de Biociências, Departamento de Botânica, Rua do Matão, 277, CEP 05508–090, São Paulo, SP, Brazil Universidade de São Paulo São Paulo Brazil

**Keywords:** *
Tanaecium
*, lianas, Lamiales, lectotype, Neotropical flora, nomenclature, taxonomy

## Abstract

*Tanaecium* Sw. emend L.G. Lohmann (Bignonieae, Bignoniaceae) is a genus of Neotropical lianas that is morphologically variable, especially in floral features. The genus is distributed from Mexico and the Antilles to Argentina, and centered in Amazonia. Here, we present an updated overview for *Tanaecium* that recognizes 21 species within the genus. Species delimitation was based on a detailed analysis of protologues and herbarium specimens, including type collections of all taxa. We present a detailed description for the genus and a key for the identification of all species. For each of the 21 species recognized, we present information on the nomenclature, phenology, habitat, distribution, and taxonomic notes. Furthermore, *Spathicalyx kuhlmannii* J.C. Gomes is transferred into *Tanaecium kuhlmannii* (J.C. Gomes) Frazão & L.G. Lohmann. A lectotype is proposed for *Tanaecium crucigerum* Seem.

## Introduction

*Tanaecium* Sw. emend L.G.Lohmann is a monophyletic genus, well supported by molecular characters (Frazão and Lohmann 2018), as well as by subulate and/or bromeliad-like prophylls of the axillary buds, a putative morphological synapomorphy (Lohmann and Taylor 2014). Species of the genus are lianas or shrubs distributed from Mexico and the Antilles to Argentina (Lohmann and Taylor 2014; Pace et al. 2016; Frazão and Lohmann 2018; Kaehler et al. 2019). The genus is centered in Amazonia, where 11 species occur (Lohmann and Taylor 2014; Frazão and Lohmann 2018; Kaehler et al. 2019). While some species show disjunct distributions (e.g., *Tanaecium duckei* A.Samp.), others are broadly distributed (e.g., *Tanaecium pyramidatum* (Rich.) L.G.Lohmann), or endemic to small geographic areas (e.g., *Tanaecium affine* (A.H.Gentry) L.G.Lohmann, *T. apiculatum* A.H.Gentry) (Lohmann and Taylor 2014; Frazão and Lohmann in prep.).

The genus was described by Swartz (1788) and originally characterized by the tubular flowers and truncate calyces. The original circumscription of *Tanaecium* included six species (see Gentry 1973; Gentry 1976), five of which remain in *Tanaecium* (i.e., *T. apiculatum*, *T. crucigerum* Seem., *T. cyrtanthum* (Mart. ex DC.) Bureau & K.Schum., *T. exitiosum* Dugand, and *T. jaroba* Sw.), while *T. nocturnum* (Barb. Rodr.) Bureau & K. Schum. was transferred to *Bignonia* L. (Lohmann and Taylor 2014). In addition to the five species originally classified as *Tanaecium*, twelve species from six previously recognized genera (i.e., *Arrabidaea* DC., *Ceratophytum* Pittier, *Pseudocatalpa* A.H.Gentry, *Paragonia* Bureau, *Periarrabidaea* A.Samp, and *Spathicalyx* J.C.Gomes) were transferred to *Tanaecium* in a revised generic classification for the whole tribe Bignonieae (Lohmann and Taylor 2014). As a result, 17 species of *Tanaecium* were recognized in the most recent synopsis of the genus (Lohmann and Taylor 2014).

Additional molecular phylogenetic studies combined with novel morphological observations indicated that *Sphingiphila tetramera* A.H.Gentry is best placed in *Tanaecium*, leading to the new combination *Tanaecium tetramerum* (A.H.Gentry) Zuntini & L.G.Lohmann (Pace et al. 2016). A new species of *Tanaecium* was subsequently described (i.e., *T. decorticans* Frazão & L.G.Lohmann) (Frazão and Lohmann 2018), while new morphological and molecular data indicates that *Tanaecium mutabile* (Bureau & K. Schum.) L.G. Lohmann is best placed within *Fridericia* Mart. emend L.G. Lohmann (Frazão and Lohmann, in prep.). More recently, Kaehler et al. (2019) transferred three species of *Fridericia* into *Tanaecium*, i.e., *Tanaecium dichotomum* (Jacq.) Kaehler & L.G.Lohmann, *T. paradoxum* (Sandwith) Kaehler & L.G.Lohmann, and *T. parviflorum* (Mart. ex DC) Kaehler & L.G.Lohmann.

Given all the recent taxonomic changes in *Tanaecium*, a new evaluation of the overall circumscription of the genus and its species is needed. Here, we present an overview for *Tanaecium*. We recognize 21 species for which we provide information on the nomenclature, synonymy, phenology, habitat, distribution, and taxonomic notes. Because *T. paradoxa* appeared within *Fridericia* in a recent phylogenetic study (Frazão and Lohmann, in prep.), we follow Lohmann and Taylor (2014) in treating this taxon as *Fridericia paradoxa* (Sandwith) L.G.Lohmann. A lectotype is proposed for *Tanaecium crucigerum* Seem., and the new combination *Tanaecium kuhlmannii* (J.C.Gomes) Frazão & L.G.Lohmann is proposed to accommodate novel morphological observations and recent phylogenetic findings (Frazão and Lohmann, in prep.).

## Material and methods

Materials from the following herbaria were studied using standard taxonomic methods (Acronyms following Thiers 2019): INPA, IAN, MG, UFACPZ, EAC, CEN, IBGE, UB, HERBAM, ESA, RBR, RB, R, SPF, SP, UEC, HRCB, CESJ, BHCB, MBM, PY, FCQ, QCNE, QCA, NY, US, MO, A, and F. Furthermore, images of specimens from AAU, B, BR, COL, G, K, L, M, and P were accessed online through Jstor Global Plants (2019) or the online database of individual herbaria. All protologues were consulted in the Peter Raven Library (Missouri Botanical Garden) or using the online database of BHL (2019). Morphological terminology used here follows Hickey (1974) for leaf venation, Radford (1986) for leaf morphology, Weberling (1989) for inflorescence morphology, Gomes-Silva (2009) for leaflet mite-domatia, Nogueira et al. (2013) for trichomes, and Lohmann and Taylor (2014) for prophyll morphology and other morphological traits. Phenology is based on data gathered from herbarium specimens. Distributions are based in data gathered from herbarium specimens and information provided in Lohmann and Taylor (2014).

## Taxonomic treatment

Tanaecium Sw., Prodr. Veg. Ind. Occ. 6: 91. 1788, emend L.G. Lohmann, Ann. Missouri Bot. Gard. 2014: 463. Type: Tanaecium jaroba Sw.

Paragonia Bureau, Bull. Soc. Bot. France 19: 17. 1872. Type: Bignonia lenta Mart. ex DC. [= Tanaecium pyramidatum (Rich.) L.G.Lohmann].

Sanhilaria Baill., Hist. Pl. 10: 27. 1888. Hilariophyton Pichon, Bull. Soc. Bot. France 92: 228. 1946. Type: Sanhilaria brasiliensis Baill. [= Tanaecium brasiliensis (Baill.) L.G.Lohmann].

Ceratophytum Pittier, J. Wash. Acad. Sci. 18: 62. 1928. Type: Ceratophytum capricorne Pittier [= Tanaecium tetragonolobum (Jacq.) L.G.Lohmann].

Periarrabidaea A. Samp., Ann. Acad. Brasil. Sci. 6: 175. 1934. Type: Periarrabidaea truncata A. Samp. [= Tanaecium truncatum (A. Samp) L.G.Lohmann].

Spathicalyx J.C.Gomes, Notul. Syst. (Paris) 15: 220. 1956. Type: Spathicalyx kuhlmannii J. C. Gomes [= Tanaecium kuhlmannii (J.C. Gomes) Frazão & L.G. Lohmann].

Pseudocatalpa A.H.Gentry, Brittonia 25(3): 241. 1973. Type: Pseudocatalpa caudiculata (Standl.) A. H. Gentry [= Tanaecium caudiculatum (Standl.) L.G.Lohmann].

*Lianas or shrubs*, without dimorphic juvenile growth; stems with four phloem wedges in cross section (without in *T. tetramerum*), solid (hollow in *T. apiculatum*); branchlets terete or tetragonal, without ridges, with or without striation, without peeling epidermis (present in *T. decorticans*), sparse or dense lenticels, with or without simple non-glandular trichomes (dendritic non-glandular trichomes in *T. xanthophyllum*); interpetiolar region with or without fields of patelliform glandular trichomes, and discontinuous interpetiolar ridges (sometimes continuous); prophylls of the axillary buds bromeliad-like and/or subulate (minute and triangular or foliaceous), without patelliform glandular trichomes (present in *T. selloi*). *Leaves* 2–3–foliolate (sometimes simple in *T. tetramerum*) with the terminal leaflet modified into a simple or trifid tendril (sometimes bifid in *T. pyramidatum*); leaflets without cartilaginous margin (present in *T. apiculatum*), secondary venation brochidodromous (craspedodromous in *T. parviflorum*). *Inflorescence* in a fascicule, raceme, thyrse or compound thyrse, terminal (sometimes axillary); calyx campanulate, cupular or tubular, bilabiate or truncate (sometimes spathaceous); corolla magenta, pink, yellow, pale yellow or white, infundibular or wide infundibular (campanulate or hypocrateriform), zygomorphic (actinomorphic in *T. tetramerum*), pentamerous (tetramerous in *T. tetramerum*), aestivation imbricate; androecium didynamous, pollen in monads, 3-colpate, psilate and microperforate (inaperturate and coarse-reticulate in *T. apiculatum*); nectar disk well-developed; gynoecium with ovary without stipe at the base, with one, two, or many series of ovules in each placenta, stigma papilose. *Capsule* elliptic or linear (linear-oblong), with or without lenticels, calyx caducous (persistent); seeds winged or wingless, with body smooth and glabrous, winged hyaline or opaque, linear, wingless corky or woody and rounded.

### Key to species of *Tanaecium*

**Table:** 

1	Branchlets thorn-tipped; terminal leaflets never replaced by a tendril; corollas hypocrateriform, 4-lobed	**19. *T. tetramerum***
–	Branchlets not thorn-tipped; terminal leaflets generally replaced by a tendril; corollas campanulate, infundibular or wide infundibular, 5-lobed	**2**
2	Leaflets with caudate apices; corollas campanulate; androecium with two fertile stamens	**4. *T. caudiculatum***
–	Leaflets without caudate apices; corollas infundibular or wide infundibular; androecium with four fertile stamens	**3**
3	Leaflets with dentate margins; calyces aristate (rarely mucronate); fruit apices caudate	**14. *T. parviflorum***
–	Leaflets without dentate margins; calyces not aristate; fruit apices not caudate	**4**
4	Leaflets with apiculate apices, with cartilagenous margins; calyces with stellate simple trichomes; pollen grains inaperturated	**2. *T. apiculatum***
–	Leaflets without apiculate apices, without cartilagenous margins; calyces without stellate simple trichomes; pollen grains colpate	**5**
5	Leaflets with emarginated membrane-like domatia; inflorescence nodes with patelliform trichome fields; corollas ≤ 2.6 cm long.	**1. *T. affine***
–	Leaflets without emarginated membrane-like domatia; inflorescence nodes without patelliform trichome fields; corollas > 2.6 cm long.	**6**
6	Stems with peeling epidermis; petiolules with arrow-shaped apices; fruits with patelliform and peltate trichomes along the margins	**7. *T. decorticans***
–	Stems without peeling epidermis; petiolules without arrow-shaped apices; fruits without patelliform and peltate trichomes along the margins	**7**
7	Leaflets 8–15 times larger than the petioles; calyces costate; corollas with cuspidate lobes	**13. *T. neobrasiliense***
–	Leaflets < 8 times larger than the petioles; calyces costate; corollas without cuspidate lobes	**8**
8	Leaflets with yellow dendritic simple trichomes; bracteoles ≥ 4:5 the flower pedicels; corollas with peltate trichomes in the ventral portion internally	**21. *T. xanthophyllum***
–	Leaflets without yellow dendritic simple trichomes; bracteoles < 4:5 the flower pedicels; corollas without peltate trichomes in the ventral portion internally	**9**
9	Leaflets with pit domatia abaxially; calyces with constriction on the basal or medial portions; corollas pale-yellow	**20. *T. truncatum***
–	Leaflets without foveolate domatia abaxially; calyces without constriction on the basal or medial portions; corollas white, pink or magenta	**10**
10	Corollas white	**11**
–	Corollas pink or magenta	**18**
11	Leaflets with pocket and tuft domatia; petioles pulvinate (rarely absent); calyces 1:3 to 2:3 the corolla tubes; ovaries with one ovule series on each placenta	**3. *T. bilabiatum***
–	Leaflets without domatia; petioles not-pulvinate; calyces ≤ 1:3 the corolla tubes; ovaries with two or many ovule series on each placenta	**12**
12	Stems with interpetiolular patelliform trichomes < 0.3 mm; inflorescences in corymbiform thyrse; corollas infundibular; fruits 4-lobed at base	**18. *T. tetragonolobum***
–	Stems with or without interpetiolular patelliform trichomes, > 0.3 mm when present; inflorescences not in corymbiform thyrse; corollas wide infundibular; fruits not 4-lobed at base	**13**
13	Leaflets with basal and suprabasal venation actinodromous; tendrils trifid; calyces spathaceous; anthers curved backwards	**14**
–	Leaflets without basal and suprabasal venation actinodromous; tendrils simple; calyces not spathaceous; anthers not curved backwards	**15**
14	Abaxial side of leaflets with patelliform trichomes ≥ 0.45 mm diam., with protrusion at the patelliform insertion; anthers ≥ 7 mm long.	**12. *T. kuhlmannii***
–	Abaxial side of leaflets with patelliform trichomes < 0.45 mm diam., without protrusion at the patelliform insertion; anthers < 7 mm long.	**9. *T. duckei***
15	Adaxial side of leaflets bullate; calyces bilabiate; anthers exserted	**10. *T. exitiosum***
–	Adaxial side of leaflets not bullate; calyces truncate; anthers sub-exserted	**16**
16	Caducuous when flowering; abaxial surface of leaflets with patelliform trichomes concentrated at base; calyces campanulate or cupular; fruits linear; seeds linear, with lateral seed body	**6. *T. cyrtanthum***
–	Not caducuous when flowering; abaxial surface of leaflets without patelliform trichomes concentrated at base; calyces cupular; fruits elliptic; seeds circular, with central seed body	**17**
17	Abaxial side of leaflets whitish-tomentose	**5. *T. crucigerum***
–	Abaxial side of leaflets glabrous or pubescent	**11. *T. jaroba***
18	Prophylls of the axillary buds foliaceous or minute and triangular; fruits with raised margins, without central ridges	**17. *T. selloi***
–	Prophylls of the axillary buds subulate or bromeliad-like; fruits with or without raised margins, with central ridges	**19**
19	Leaflets with margin curvature revolute; fruits linear-oblong; seeds with vestigial wings; distributed along riparian areas in the Amazon	**16. *T. revillae***
–	Leaflets with margin curvature flat; fruits linear; seeds with well-developed wings; distributed in all habitat types throughout the Neotropics	**20**
20	Petioles with patelliform trichomes at apices; tendrils bifid or trifid; fruits inflated and lenticellated	**15. *T. pyramidatum***
–	Petioles without patelliform trichomes at apices; tendrils simple; fruits flattened and not lenticellated	**8. *T. dichotomum***

#### 1. Tanaecium affine

Taxon classificationPlantaeLamialesBignoniaceae

(A.H.Gentry) L.G.Lohmann. Ann. Missouri Bot. Gard. 99: 464. 2014.

B9A27B7E-A5A7-545D-B541-CE7A76655BAD

Arrabidaea affinis A.H.Gentry, Novon 2(2): 159. 1992. Type: Ecuador. Sucumbios: Lake Agrio, banks of lake, 250 m, 0°6'45.28"N, 76°54'42.81"W, 1 Apr. 1980, J. Brandbyge and E. Asanza 30393 (holotype, MO [MO-083145]!; isotypes, AAU image!, AAU photo at MO!, NY [NY00000106]!).

##### Habitat and distribution.

*Tanaecium affine* is known from humid forests with rich soils, although it has been collected in primary and secondary forests with lateritic soil in Peru (Loreto, Mayanas). It is native from Bolivia (La Paz), Colombia (Antioquia, Boyaca), Ecuador (Napo, Pastaza, Sucumbíos), and Peru (Amazonas, Junín, Loreto, Pasco, Puno).

##### Phenology.

Flowering: February to April, September and November; fruiting: February to December.

##### Notes.

This species is morphologically similar to *Fridericia florida* but differs by the bilabiate calyces, stems with conspicuous patelliform trichomes in the interpetiolar region, and occurrence in rich soils (Gentry 1992). In addition, *T. affine* can also be recognized by the numerous peltate trichomes distributed throughout the leaflets, emarginated membrane-like domatia, and fields of patelliform trichomes that cover the inflorescence nodes. *Tanaecium affine* shares vegetative traits with *Tanaecium tetragonolobum*, a sympatric species (Tab. 1). However, *T. tetragonolobum* can be differentiated by the glabrous leaflets (vs. leaflets covered with peltate trichomes in *T. affine*), petioles longer than petiolules (vs. petioles shorter than petiolules in *T. affine*), and subulate prophylls of the axillary buds (vs. bromeliad-like prophylls of the axillary buds in *T. affine*).

**Table 1.** Characters useful to recognize *Tanaecium* species.

*Tanaecium* species		Branchlet section	Interpetiolar glandular field	Prophylls of the axillary buds	Tendril type	Inflorescence type	Calyx shape	Calyx aperture	Corolla color	Corolla mouth color	Corolla shape	Ovules series	Fruit shape	Seeds wings
1.	*Tanaecium affine*	terete or tetragonal	present	subulate or bromeliad-like	simple	compound thyrse	campanulate	bilabiate	white	white	infundibular	one	linear	well-developed
2.	*Tanaecium apiculatum*	terete	absent	subulate or bromeliad-like	absent	raceme	tubular	truncate	white	white	wide infundibular	many	-	-
3.	*Tanaecium bilabiatum*	terete	absent	subulate or bromeliad-like	simple	thyrse	campanulate or tubular	bilabiate	white	yellow	infundibular	one	linear	vestigial
4.	*Tanaecium caudiculatum*	tetragonal	absent	subulate or bromeliad-like	simple	thyrse	campanulate	truncate	pale yellow	white	campanulate	one	linear	well-developed
5.	*Tanaecium crucigerum*	terete	present	minute and triangular or bromeliad-like	simple	thyrse	cupular	truncate	white	white	wide infundibular	many	elliptic	absent
6.	*Tanaecium cyrtanthum*	terete	present	minute and triangular or bromeliad-like	simple	thyrse	cupular	truncate	white	white	wide infundibular	many	linear	well-developed
7.	*Tanaecium decorticans*	terete	present	subulate	trifid	thyrse	campanulate or cupular	truncate	pink	white	infundibular	one	linear	well-developed
8.	*Tanaecium dichotomum*	terete	present or absent	subulate or bromeliad-like	simple	thyrse	campanulate	bilabiate	pink	white	campanulate or infundibular	one	linear	well-developed
9.	*Tanaecium duckei*	terete	absent	subulate	trifid	thyrse	tubular	oblique	white	white	wide infundibular	many	linear	well-developed
10.	*Tanaecium exitiosum*	terete	absent	subulate	simple	thyrse	campanulate	bilabiate	white	white	wide infundibular	-	-	-
11.	*Tanaecium jaroba*	terete	present	minute and triangular or bromeliad-like	simple	thyrse	campanulate	truncate	white	white	wide infundibular	many	elliptic	absent
12.	*Tanaecium kuhlmannii*	terete	absent	subulate	trifid	thyrse	tubular	oblique	white	white	wide infundibular	many	linear	well-developed
13.	*Tanaecium neobrasiliense*	terete	absent	subulate or bromeliad-like	trifid	compound thyrse	campanulate	truncate	magenta	-	infundibular	two	linear	well-developed
14.	*Tanaecium parviflorum*	terete or tetragonal	absent	subulate or bromeliad-like	simple	thyrse	campanulate	truncate	white	yellow	infundibular	two	linear	well-developed
15.	*Tanaecium pyramidatum*	terete	absent	subulate or bromeliad-like	bifid or trifid	compound thyrse	campanulate	bilabiate or truncate	pink or magenta	white	infundibular	one	linear	well-developed
16.	*Tanaecium revillae*	terete	absent	subulate or bromeliad-like	simple	thyrse	campanulate	bilabiate	pink	white	infundibular	one	linear-oblong	vestigial
17.	*Tanaecium selloi*	terete	absent	minute and triangular or foliaceous	simple	thyrse	campanulate	bilabiate	pink	white	infundibular	one	linear	well-developed
18.	*Tanaecium tetragonolobum*	terete or tetragonal	present	subulate	simple	thyrse	cupular	truncate	white	yellow	infundibular	many	linear	well-developed
19.	*Tanaecium tetramerum*	terete	absent	subulate or bromeliad-like	absent	fascicule	tubular	truncate	white	white	hypocrateriform	two	elliptic	well-developed
20.	*Tanaecium truncatum*	terete	present	subulate or bromeliad-like	trifid	thyrse	campanulate	oblique or truncate	pale yellow	yellow	infundibular	two	linear	well-developed
21.	*Tanaecium xanthophyllum*	terete	present	minute and triangular or bromeliad-like	trifid	compound thyrse	campanulate	bilabiate	yellow	yellow	infundibular	two	linear	well-developed

#### 2. Tanaecium apiculatum

Taxon classificationPlantaeLamialesBignoniaceae

A.H.Gentry, Ann. Missouri Bot. Gard. 63(1): 58, fig. 4. 1976.

7EBABC2C-E60E-51A4-9882-B188CE5616FA

##### Type.

Venezuela. Monagas: Caicara, 15 May 1952, F. D. Smith 226 (holotype, US [US-2121468]!; isotype, US!, US photo at MO [MO-067514]!, [MO-067514]!)

##### Habitat and distribution.

*Tanaecium apiculatum* is known only from the type location, Caicara, Venezuela.

##### Phenology.

Flowering: May; fruiting (immature): May.

##### Notes.

This species shares wide infundibular corollas with *T. crucigerum*, *T. cyrtanthum*, *T. duckei*, *T. exitiosum*, *T. kuhlmannii*, and *T. jaroba*, but can be differentiated from these taxa by the leaflets with apiculate apices and cartilaginous margins, and tubular calyces with stellate simple trichomes (Tab. 1). Out of these species, *Tanaecium crucigerum* and *T. jaroba* are the only ones that also occur in Venezuela. These species can be differentiated from *T. apiculatum* by the abaxial surface whitish-tomentose in *T. crucigerum* and abaxial surface glabrous or pubescent in *T. jaroba* (vs. abaxial surface glabrous in *T. apiculatum*).

#### 3. Tanaecium bilabiatum

Taxon classificationPlantaeLamialesBignoniaceae

(Sprague) L.G.Lohmann, Nuevo Cat. Fl. Vasc.Venezuela 274. 2008.

2A487B19-6954-5B7F-8AE1-306D2BCC0377

Fig. 1A, L

Memora bilabiata Sprague, Bull. Herb. Boissier (ser. 2) 6: 375. 1906.
Adenocalymma bilabiatum (Sprague) Sandwith, Recueil Trav. Bot. Néerl. 34: 213. 1937. Type: Brazil. Amazonas: Manaus, s.d., R. Spruce 1783 (holotype, K [K000492969] image!).

##### Habitat and distribution.

*Tanaecium bilabiatum* grows in wet, flooded, riparian vegetation, or Amazonian lowlands. It occurs in Bolivia (Beni, Pando), Brazil (Acre, Amapá, Amazonas, Pará, Roraima), Colombia (Amazonas, Arauca, Guainía), French Guyana, Guyana, Peru (Madre de Dios, Loreto), Suriname (Sipaliwini, Nickerie), and Venezuela (Amazonas, Apure, Bolívar, Delta Amacuro, Guárico, Monagas, Sucre).

##### Phenology.

Flowering: February to November; fruiting: December to October.

##### Notes.

*Tanaecium bilabiatum* is easily differentiated from other *Tanaecium* species by the pulvinated petioles (typical of *Adenocalymma* but usually lacking in *Tanaecium* and other Bignonieae; Lohmann and Taylor 2014), large bilabiate calyces, covering 1:3 to 2:3 of the corolla tube, white corollas with yellow mouths, oblong and flattened fruits, and seeds with vestigial wings (rarely well-developed) (Tab. 1).

**Figure 1.**
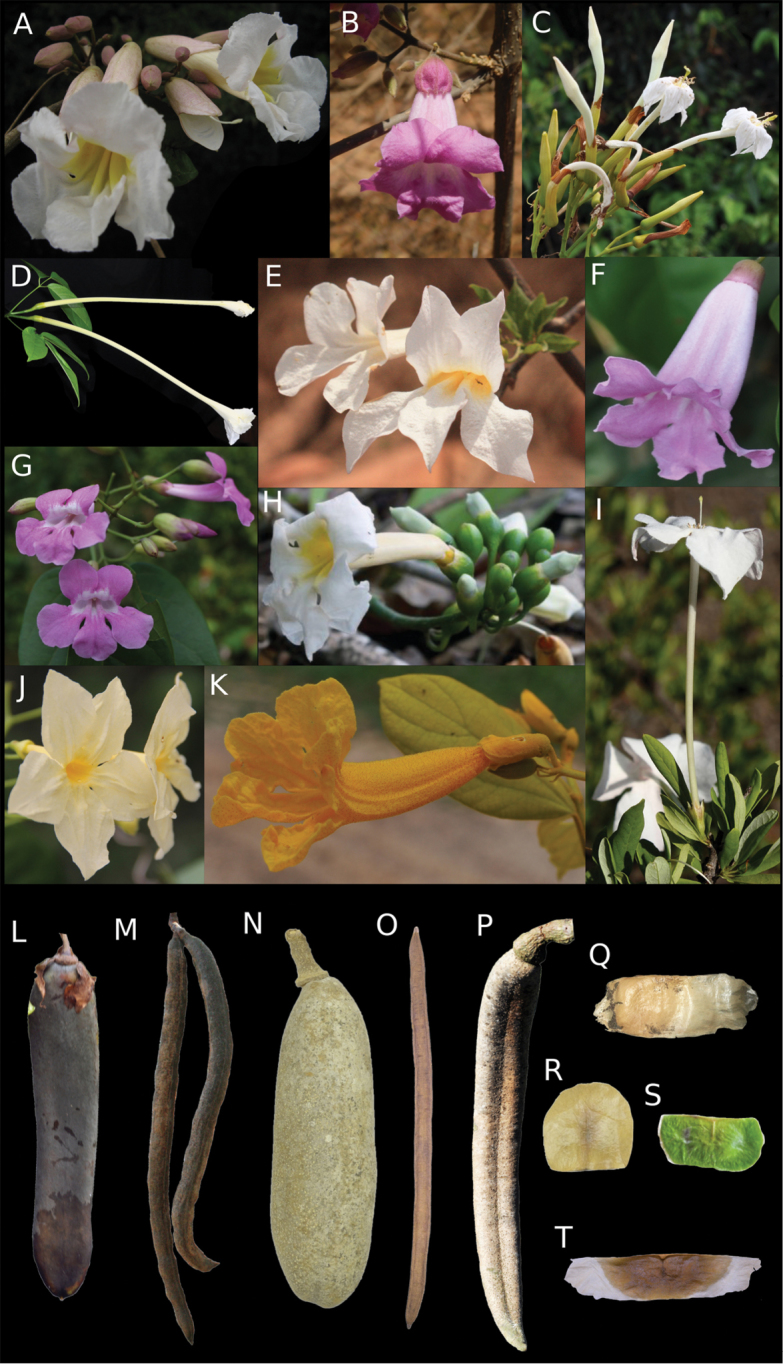
Morphological diversity of *Tanaecium*. **A–K** Flowers **A***T. bilabiatum***B***T. dichotomum***C***T. duckei***D***T. jaroba***E***T. parviflorum***F***T. pyramidatum***G***T. revillae***H***T. tetragonolobum***I***T. tetramerum***J***T. truncatum***K***T. xanthophyllum.***L–P** Fruits **L***T. bilabiatum***M***T. cyrtanthum***N***T. jaroba***O***T. selloi***P***T. tetragonolobum*. **Q–T** Seeds **Q***T. cyrtanthum***R***T. jaroba***S***T. revillae***T***T. selloi*. Photos by A. Frazão, except **A** by B. Gomes **B** by R. Lopes **E, M** by C. Siniscalchi **G** by E. Kataoka **H** by Stevens **I** by Parada-Gutierrez **J** by L.H.M. Fonseca.

#### 4. Tanaecium caudiculatum

Taxon classificationPlantaeLamialesBignoniaceae

(Standl.) L.G.Lohmann, Ann. Missouri Bot. Gard. 99(3): 464.

E6932D07-9519-5C45-AAA1-3B2D397A25C9

Petastoma caudiculatum Standl. Publ. Field Mus., Bot. 11(4): 141. 1932.
Pseudocatalpa caudiculata (Standl.) A.H.Gentry, Brittonia 25(3): 241. 1973. Type: Belize. Nine Mile, Stann Creek Railway, 30 m, 22 Mar. 1932, W. A. Schipp S–297 (holotype, F!).

##### Habitat and distribution.

*Tanaecium caudiculatum* is restricted to Central America. It is known from wet forests that grow in the mountains and sea level in Belize (Belize, Cayo, Stann Creek, Toledo), Guatemala (Alta Vera Cruz), and Mexico (Chiapas, Oaxaca).

##### Phenology.

Flowering: March to May, July to September; fruiting: April, June, and August.

##### Notes.

*Tanaecium caudiculatum* differs from other species in the genus by the caudate leaflets, simple tendrils that bear hooks (otherwise only found in the trifid tendrilled *Dolichandra*; Lohmann and Taylor 2014; Fonseca et al. 2017), foliaceous inflorescence bracts, stipitate glandular trichomes in the internal ventral surface of the corolla tubes, androecium with only two fertile stamens, and fruits with sinuous margins (Tab. 1).

#### 5. Tanaecium crucigerum

Taxon classificationPlantaeLamialesBignoniaceae

Seem., Bonplandia (Hannover) 4: 127. 1856.

AE869355-597D-50F2-842D-1EBD62ED7496

##### Type.

Lesser Antilles. Dominica, sin. loc., s. d., J. Imray 94 (lectotype, designated here, K [K000449535] image!).

##### Habitat and distribution.

*Tanaecium crucigerum* occurs in wet forests in the Lesser Antilles (Dominica, Martinique), Trinidad and Tobago, Costa Rica (Limón), and Venezuela (Anzoátegui, Apure, Cojedes, Delta Amacuro, Guárico, Portuguesa).

##### Phenology.

Flowering: April to July, and October; fruiting: February, April to July, and October to November.

##### Notes.

Like Lohmann and Taylor (2014), we were also unable to locate the lectotype of *T. crucigerum* selected by Howard (1989: 334), the collection *J. Imray 95* supposedly deposited at K. This collection is thus presumed lost. As such, we select another Imray collection from Dominica studied by Seemmann (1856) deposited at K as lectotype. We selected the material *J. Imray 94* as lectotype due to high quality of this material.

This species is morphologically most similar to *T. jaroba*, sharing many characters such as the simple tendrils, wide infundibular corollas, and wingless seeds (Tab. 1). *Tanaecium crucigerum* differs from *T. jaroba* by the whitish-tomentose leaflets on the abaxial surface (vs. glabrous or pubescent leaflets on the abaxial surface in *T. jaroba*).

#### 6. Tanaecium cyrtanthum

Taxon classificationPlantaeLamialesBignoniaceae

(Mart. ex DC.) Bureau & K.Schum, Fl. Bras. 8(2): 186. 1896.

4B2C5FF2-6162-5E5C-ADC6-962821A5D4F1

Fig. 1M, Q.

Tecoma cyrtantha Mart. ex DC., in A. DC., Prodr. 9: 218. 1845. Type: Brazil. Bahia: Pão d’Espinho, caatinga, Oct., C.F.P. von Martius 1860 (holotype, M [M0088980]!; isotype, G-DC!).

##### Habitat and distribution.

*Tanaecium cyrtanthum* is distributed in dry forests, caatinga, cerrado and chaco in Bolívia (Santa Cruz, Tarija), Brazil (Bahia, Ceará, Goiás, Mato Grosso do Sul, Pernambuco, Rio Grande do Norte), and Paraguay (Alto Paraguay, Amambay, Concepción, San Pedro).

##### Phenology.

Flowering: September to January and April; fruiting: April to August and October.

##### Notes.

This species is generally caducous when flowering, and produces new leaves when fruiting. The tendril is simple and the leaflets have patelliform trichomes concentrated at the base abaxially. The calyces are campanulate or cupular, while the fruits are linear and inflated, bearing linear seeds, with a lateral seed body (Tab. 1).

#### 7. Tanaecium decorticans

Taxon classificationPlantaeLamialesBignoniaceae

Frazão & L.G.Lohmann, Pl. Syst. Evol. 304: 1248. fig. 2. 2018.

16214D2B-0A88-5FCE-B9C9-D826D425CDB3

##### Type.

Brazil. Pará: Belterra, Entrada da estrada de Aramanaí para Pindobal, próximo a Fazenda São Sebastião, 41 m a. s. l., 2°38'24.7"S, 54°59'06.6"W, 20 Sep 2015, A. Frazão 210 (holotype: SPF!; isotype: RB!, MO!).

##### Habitat and distribution.

*Tanaecium decorticans* is known from the Brazilian Amazon (Pará, Maranhão).

##### Phenology.

Flowering: February and September; fruiting: September and December.

##### Notes.

This species is morphologically most similar to *T. pyramidatum*, sharing characters such as the subulate prophylls, infundibular corolla with white mouth, and linear fruits (Tab. 1). However, *T. decorticans* can be differentiated from *T. pyramidatum* by the stems with peeling epidermis (vs. stems without peeling epidermis in *T. pyramidatum*), petiolules with arrow-shaped apices (vs. lacking in *T. pyramidatum*), and fruits flattened with glandular patelliform and peltate trichomes along the margins (vs. fruits inflated without glandular patelliform and peltate trichomes along the margins in *T. pyramidatum*) (Frazão and Lohmann 2018) (Tab. 1).

#### 8. Tanaecium dichotomum

Taxon classificationPlantaeLamialesBignoniaceae

(Jacq.) Kaehler & L.G.Lohmann, in press

75075504-7855-5C97-87C1-636D38655359

* Fig. 1B

Bignonia dichotoma Jacq. Enum. Syst. Pl. 25. 1760 [also in Select. stirp. amer. hist. 183, 1763].
Fridericia dichotoma (Jacq.) L.G. Lohmann, Ann. Missouri Bot. Gard. 99: 436. 2014. Type: Colombia. Magdalena: Cartagena, not located.

##### Habitat and distribution.

*Tanaecium dichotomum* is commonly found in dry to humid forests in Argentina (Chaco, Corrientes, Formosa, Jujuy, Misiones, Salta), Belize (Cayo), Bolivia (Beni, Chuquisaca, La Paz, Pando, Santa Cruz, Tarija), Brazil (Acre, Alagoas, Amapá, Amazonas, Bahia, Ceará, Distrito Federal, Goiás, Maranhão, Mato Grosso, Mato Grosso do Sul, Minas Gerais, Pará, Paraíba, Pernambuco, Piauí, Rio de Janeiro, Rio Grande do Sul, Rondônia, Roraima, Santa Catarina, São Paulo, Tocantins), Colombia (Amazonas, Atlántico, Bolívar, César, Chocó, Huila, La Guajira, Magdalena, Meta, Sucre, Tolima), Costa Rica (Guanacaste, Puntarenas), Ecuador (Guayas, Napo), French Guiana, Guyana (Essequibo, Rupununi), Mexico (Chiapas, Colima, Guerrero, Jalisco, Mexico, Oaxaca, Veracruz), Nicaragua (Boaco, Chontales, Granada, Matagalpa, Nueva Segovia, Río San Juan), Panama (Canal Area, Panama, Los Santos), Paraguay (Alto Paraguay, Amambay, Boquerón, Central, Chaco, Concepción, Cordillera, Ñeembucú, Nueva Asuncion, Paraguarí, Presidente Hayes, San Ramon), Peru (Cusco, Loreto, Madre de Dios, Piura, San Martín, Tumbes, Ucayali), and Venezuela (Amazonas, Anzoátegui, Apure, Aragua, Barinas, Bolívar, Carabobo, Cojedes, Distrito Federal, Falcón, Guárico, Lara, Mérida, Miranda, Monagas, Nueva Esparta, Portuguesa, Sucre, Táchira, Trujillo, Yaracuy, Zulia).

##### Phenology.

Flowering: January to December; fruiting: January to December.

##### Notes.

This species is widespread through the Neotropics, where it is found in many vegetation types. The species encompasses an enormous degree of morphological variation, representing a species complex. Detailed morphological and molecular studies are necessary to sort out the patterns of variation and identify putative cryptic species.

*Tanaecium dichotomum* shares many morphological traits with *T. selloi* and *T. revillae* (e.g., tuft domatia in the abaxial side of leaflets, bilabiate calyces), and *T. pyramidatum* (e.g., thyrsoid inflorescences, pink corollas with white mouths). However, *T. dichotomum* differs from these species by the bilabiate and cuspidate calyces, stems with patelliform glandular trichomes between the petioles, and flattened fruits without raised margins or a conspicuous central ridge (Tab. 1).

#### 9. Tanaecium duckei

Taxon classificationPlantaeLamialesBignoniaceae

A.Samp., Ann. Acad. Brasil. Sci. 7: 125. 1935.

D7944EDA-55EE-574F-B237-F5FC6EF961F4

Fig. 1C

Spathicalyx duckei (A.Samp.) A.H.Gentry, Phytologia 35(3): 194. 1977. Type: Brazil. Pará: Óbidos, 21 July 1918, A. Ducke s.n. (holotype, MG!; isotypes, MO [MO-077163]!, R!, RB [RB00536923]!, US [US00125782]!).

##### Habitat and distribution.

*Tanaecium duckei* grows in Amazonian forests with sandy soils and canga vegetation. It occurs in Brazil (Acre, Amazonas, Pará, Mato Grosso), Colombia (Amazonas), and Peru (Loreto, Pasco).

##### Phenology.

Flowering: July and September to October; fruiting: September.

##### Notes.

This species differs from other species of *Tanaecium* by the spathaceous calyces, reflexed anthers, and vegetative structures covered by stipitate glandular trichomes. It is morphologically most similar to *T. kuhlmannii*, with which it shares trifid tendrils and spathaceous calyces (Tab. 1). It is possible to separate *T. duckei* from *T. kuhlmannii* by the lack of patelliform glandular trichomes along the tertiary veins (vs. present in *T. kuhlmannii*), green fruits with sparse stipitate glandular trichomes (vs. yellow fruits covered by stipitate glandular trichomes in *T. kuhlmannii*), smaller anthers with 4.17–4.34 mm long (vs. larger anthers with 7.0–10.0 mm long in *T. kuhlmannii*), and stamens inserted at the same height (vs. stamens inserted at two different heights in *T. kuhlmannii*) (Tab. 1).

#### 10. Tanaecium exitiosum

Taxon classificationPlantaeLamialesBignoniaceae

Dugand, Caldasia 1(5): 31, fig. 1. 1942.

BBAD09D1-B95F-50E7-8BA8-DDC76EAB3E33

##### Type.

Colombia. Santander: Barrancabermeja, 50 m, 5 Apr. 1942, R. Mora s.n. (holotype, COL [COL000004390]!; isotype, COL [COL000004389]!).

##### Habitat and distribution.

*Tanaecium exitiosum* is endemic to wet forest vegetation from Colombia (Caldas, Santander).

##### Phenology.

Flowering: March to April and December; fruiting: unknown.

##### Notes.

This species shares wide infundibular white flowers with *T. apiculatum*, *T. crucigerum*, *T. cyrtanthum*, *T. duckei*, *T. kuhlmannii*, and *T. jaroba*, from which it differs by the leaflets bullate adaxially, calyces campanulate and bilabiate, and anthers exserted (Tab. 1). Among the most similar species, *Tanaecium exitiosum* is only sympatric with *T. jaroba*, from which it can be distinguished by the lack of interpetiolar glandular fields (vs. present in *T. jaroba*), bilabiate calyces (vs. truncate calyces in *T. jaroba*), and exserted anthers (vs. sub-exserted anthers in *T. jaroba*) (Tab. 1).

#### 11. Tanaecium jaroba

Taxon classificationPlantaeLamialesBignoniaceae

Sw., Prodr. 92: 1788.

E4CB5244-3A6A-5AC9-AE86-878ECCF5A85D

Fig. 1D, N, R

##### Type.

Jamaica, s. loc., s.d., O. Swartz s.n. (holotype, S not seen).

##### Habitat and distribution.

*Tanaecium jaroba* grows in flooded and swampy forests (Gentry 1997) in Bolivia (Beni, La Paz), Brazil (Acre, Amazonas, Mato Grosso, Mato Grosso do Sul, Pará, Rondônia, Roraima), Colombia (Amazonas, Antioquia, Atlántico, Bolívar, Caquetá, La Guajira, Magdalena, Sucre), Costa Rica (Limón), Ecuador (Napo, Orellana), French Guiana (Cayenne), Guyana, Lesser Antilles (Jamaica, St. Vincent), Panamá (Panamá), Peru (Loreto, Madre de Dios, Ucayali), Trinidad and Tobago, and Venezuela (Amazonas, Apure, Bolívar, Carabobo, Delta Amacuro, Guárico, Zulia).

##### Phenology.

Flowering: April to August and November to December; fruiting: March to August and December.

##### Notes.

This species has the longest wide infundibular white flowers in the whole tribe Bignonieae, with corollas up to 35 cm long (Gentry 1997, Howard 1989). It is most morphologically similar to *T. crucigerum*, with which it shares ellipsoid fruits that bear wingless woody seeds (Tab. 1). *Tanaecium jaroba* differs from *T. crucigerum* by the glabrous or pubescent leaflets abaxially (vs. whitish-tomentose leaflets abaxially in *T. crucigerum*).

#### 12. Tanaecium kuhlmannii

Taxon classificationPlantaeLamialesBignoniaceae

(J.C.Gomes) Frazão & L.G.Lohmann comb. nov.

5036D8BF-C961-5F12-97A7-9C25DF1F19FD

Basionym: Spathicalyx kuhlmannii J.C. Gomes, Arq. Srv. Fl., Rio de Janeiro 10: 200. 1956. Type: Brazil. Rio de Janeiro: Sumaré, 5 Dec. 1932, J.G. Kuhlmann s.n. (holotype, RB!; isotype, SPF!, K image!, MO!).

##### Habitat and distribution.

*Tanaecium kuhlmannii* is known from only a few localities within humid formations of the Atlantic Forest of Brazil (Minas Gerais, Rio de Janeiro).

##### Phenology.

Flowering: December; fruiting: January.

##### Notes.

Gomes (1956) originally described this species as *Spathicalyx kuhlmannii* J.C.Gomes, but Gentry (1977) synonymized it with *Spathicalyx duckei* (A.Samp.) A.H.Gentry. More recently, Lohmann and Taylor (2014) synonymized *Spathicalyx* with *Tanaecium* and recognized a single species, *Tanaecium duckei* (A. Samp.) L.G.Lohmann, following Gentry (1977). A detailed study of these taxa showed that apart from the allopatric distribution (*T. duckei* is restricted to the Amazon, while *T. kuhlmannii* is restricted to the Atlantic Forest of Brazil), *T. kuhlmannii* can be distinguished by the patelliform glandular trichomes along the tertiary veins of leaflets (vs. absent in *T. duckei*), and the ferrugineous stipitate glandular trichomes that cover the fruit surface (vs. ferrugineous stipitate glandular trichomes lacking in *T. duckei*). Furthermore, *T. kuhlmannii* has leaflets with patelliform trichomes ≥ 0.45 mm in diameter abaxially (vs. leaflets with patelliform trichomes < 0.45 mm in diameter abaxially in *T. duckei*), that also show a protrusion at the patelliform insertion (vs. without protrusion at the patelliform insertion in *T. duckei*), and anthers ≥ 7 mm long. (vs. anthers < 7 mm long in *T. duckei*). Based on these morphological features and distribution data, we here recognize both taxa as separate and propose the new combination *Tanaecium kuhlmannii* (J.C.Gomes) Frazão & L.G.Lohmann (Tab. 1).

#### 13. Tanaecium neobrasiliense

Taxon classificationPlantaeLamialesBignoniaceae

L.G.Lohmann, Ann. Missouri Bot. Gard. 99(3): 465. 2014.

091B88DF-B0D2-53DD-B8AD-82490E844CEC

Sanhilaria brasiliensis Baill., Hist. Pl. 10: 27. 1888.
Paragonia brasiliensis (Baill.) A. H. Gentry, Ann. Missouri Bot. Gard. 63(1): 70. 1976. Type: Brazil. Minas Gerais: Itabira, 1816–1821, A.St. Hilaire 745 (holotype, P [P00458597]!; isotypes, P [P00468598]!, F [F0092570]!).

##### Habitat and distribution.

*Tanaecium neobrasiliense* is found in caatinga and cerrado in eastern Brazil (Bahia, Ceará, Distrito Federal, Minas Gerais).

##### Phenology.

Flowering: November to January; fruiting: January to April and June.

##### Notes.

This species is generally confused with *T. pyramidatum* due to its pink corollas. However, it can be differentiated from *T. pyramidatum* by the leaflets 8–15 times longer than the petiole, costate calyces, and corollas with cuspidate lobes. The prophylls of the axillary buds are subulate or bromeliad-like, positioned in an acute angle in relation to the stems (vs. straight angle in *T. pyramidatum*) (Tab. 1).

#### 14. Tanaecium parviflorum

Taxon classificationPlantaeLamialesBignoniaceae

(Mart. ex DC.) Kaehler & L.G.Lohmann, in press

F4781BC8-75BE-5412-A99C-31185EA4641D

** Fig. 1E

Pithecoctenium parviflorum Mart. ex DC. in A.DC. Prodr 9: 197. 1845.
Arrabidaea parviflora (Mart. ex DC.) Bureau & K.Schum. in Fl. Bras. 8(2): 53. 1896.
Fridericia parviflora (Mart. ex DC.) L.G.Lohmann, Ann. Missouri Bot. Gard. 99(3): 441. 2014. Type: Brazil. Bahia, Vale do Rio das Contas, October 1818, C.F.P. von Martius s.n. (lectotype, selected by Lohmann and Taylor 2014, M [M0086353]!).

##### Habitat and distribution.

*Tanaecium parviflorum* occurs in caatinga vegetation from eastern Brazil (Bahia, Ceará, Minas Gerais, Paraíba, Pernambuco), and is also found disjunctly in Mato Grosso do Sul, in an area with drained soil.

##### Phenology.

Flowering: December to February and April; fruiting: February to March and November to December.

##### Notes.

*Tanaecium parviflorum* can be distinguished from all other species of the genus by the dentate leaflet margins, calyces aristate (rarely mucronate), and fruit apices caudate. Like *T. cyrtanthum* and *T. tetramerum*, this species is also caducous when flowering. However, *T. parviflorum* differs from these two species by the strongly compressed corollas (Tab. 1).

#### 15. Tanaecium pyramidatum

Taxon classificationPlantaeLamialesBignoniaceae

(Rich.) L.G.Lohmann, Nuevo Cat. Fl. Vasc. Venezuela 274. 2008.

2CD95E56-BB90-527C-9B45-EA6AE137067C

Fig. 1F

Bignonia pyramidata Rich., Actes Soc. Hist. Nat. Paris 1: 110. 1792.
Tabebuia pyramidata (Rich.) DC., in A. DC., Prodr. 9: 214. 1845.
Paragonia pyramidata (Rich.) Bureau, Konigl. Danske Vidansk. Selsk. Skr., Naturivdensk. Math. Afd., ser. 6, 6: 422. 1892. Type: French Guiana. Cayenne, s. d., J. B. Leblond 292 (holotype, P-LA [P00358235]!; isotype, P-LA [P00358236]!).

##### Habitat and distribution.

*Tanaecium pyramidatum* is widespread throughout the Neotropics, where it is found in dry and wet vegetation in Belize (Cayo, Toledo, Stann Creek, Belize, Orange Walk, Corozal), Bolivia (Beni, Cochabamba, La Paz, Pando, Santa Cruz), Brazil (Acre, Amapá, Amazonas, Bahia, Ceará, Distrito Federal, Goiás, Maranhão, Mato Grosso, Mato Grosso do Sul, Minas Gerais, Pará, Paraíba, Paraná, Pernambuco, Piauí, Rio de Janeiro, Rio Grande do Sul, Rondônia, Roraima, Santa Catarina, São Paulo, Tocantins), Colombia (Amazonas, Antioquia, Atlántico, Boyacá, Caquetá, Chocó, Córdoba, Cundinamarca, Guaviare, Magdalena, Meta, Nariño, Putumayo, Santander, Valle del Cauca, Vaupés), Costa Rica (Alajuela, Guanacaste, Heredia, Limón, Puntarenas, San José), Ecuador (El Oro, Esmeraldas, Guayas, Loja, Los Ríos, Manabí, Napo, Pastaza, Pichincha, Sucumbíos, Zamora-Chinchipe), El Salvador (Ahuachapán, La Libertad, Usulután), Guatemala (Alta Verapaz, Izabal, Petén), French Guiana (Cayenne, Saint-Laurent-du-Maroni), Guyana (East Berbice, Rupununi, West Demerara), Honduras (Colón, El Paraíso, Gracias a Dios, Islas de la Bahía, Olancho, Yoro), Mexico (Campeche, Chiapas, Colima, Oaxaca, Quintana Roo, Tabasco, Veracruz), Nicaragua (Atlántico Norte, Atlántico Sur, Chontales, Jinotega, Matagalpa, Río San Juan, Rivas), Panama (Bocas del Toro, Canal Area, Chiriquí, Coclé, Colón, Darién, Herrera, Los Santos, Panamá, San Blas, Veraguas), Peru (Amazonas, Cusco, Huánuco, Junín, Loreto, Madre de Dios, Pasco, Puno, San Martín, Ucayali), Suriname (Nickerie, Saramacca, Sipaliwini), Trinidad and Tobago, and. Venezuela (Amazonas, Anzoátegui, Apure, Barinas, Bolívar, Delta Amacuro, Distrito Federal, Falcón, Lara, Miranda, Monagas, Portuguesa, Sucre, Yaracuy, Zulia),

##### Phenology.

Flowering: January to December; fruiting: January to December.

##### Notes.

This species can be distinguished from other *Tanaecium* species by the petioles with patelliform trichomes at the apices, subulate prophylls of the axillary buds, fruits lenticellated, linear, and inflated. Despite that, *T. pyramidatum* is extremely variable morphologically. For example, populations from the Brazilian dry forests and cerrados have pubescent leaflets abaxially, a feature not found in any other population of this species. On the other hand, populations from Mexico are strongly covered by lenticels. Both of these features are found exclusively in these populations. Additional studies of *T. pyramidatum*, including phylogeographic studies based on a broad sampling of individuals collected throughout the range of this species, are necessary to identify putative cryptic species (Tab. 1).

#### 16. Tanaecium revillae

Taxon classificationPlantaeLamialesBignoniaceae

(A.H.Gentry) L.G.Lohmann, Ann. Missouri Bot. Gard. 99(3): 466.

C65D3665-71AC-5353-BCC4-47A3A5DF19E2

Fig. 1G, S

Arrabidaea revillae A.H.Gentry, Ann. Missouri Bot. Gard. 65(2): 726, fig. 1. 1978 [1979]. Type: Peru. Loreto: Maynas, distr. Pebas, Río Yahuasyacu, afluente del Río Ampiyacu, 18 Jul. 1976, J. Revilla 718 (holotype, MO [MO-086234]!; isotypes, COL [COL000004271]!, F–1797223!, NY [00313111]!, AMAZ not seen, USM not seen)

##### Habitat and distribution.

*Tanaecium revillae* occurs in riparian vegetation and permanently flooded forest of the Amazon region. It occurs in Brazil (Amazonas, Pará, Roraima), Colombia (Caquetá), Guyana (Upper Takutu-Upper Essequibo), Peru (Loreto), and Suriname (Sipaliwini).

##### Phenology.

Flowering: January, April, June to September and November; fruiting: July to August.

##### Notes.

This species is well characterized morphologically and can be separated from other species of *Tanaecium* by the elliptic to ovate leaflets with cuspidate apices, tuft domatia in the abaxial surface of leaflets, fruits linear-oblong covered with peltate and patelliform glandular trichomes, and flat seeds with vestigial wings (Tab. 1).

#### 17. Tanaecium selloi

Taxon classificationPlantaeLamialesBignoniaceae

(Spreng.) L.G.Lohmann, Nuevo Cat. Fl. Vasc. Venezuela 274. 2008.

2F71BFB2-0A81-5706-A526-494E50176AEE

Fig. 1O, T

Bignonia selloi Spreng., Syst. Veg. 2: 831. 1825.
Arrabidaea selloi (Spreng.) Sandwith, Kew Bull. 8(4): 461. 1953 [1954]. Type: Brazil. Sin. loc., 1840, F. Sellow s. n. (holotype, B destroyed; lectotype, selected by Arbo 2017 in K [K000402778] image!; isolectotypes, BR [BR0000008764805] image!, G [G00133280] image!, K [K000402780] image!, L [L0412987] image!).
Bignonia coriacea Sellow ex Steud. Nomencl. Bot., ed. 2, 1: 204. 1840.

##### Habitat and distribution.

This species is found in semi-deciduous dry or wet vegetation in Argentina (Chaco, Corrientes, Jujuy, Misiones, Salta), Bolivia (Chuquisaca, La Paz, Santa Cruz, Tarija), Brazil (Bahia, Ceará, Distrito Federal, Espírito Santo, Goiás, Mato Grosso do Sul, Minas Gerais, Paraíba, Paraná, Pernambuco, Rio de Janeiro, Rio Grande do Sul, Roraima, Santa Catarina, São Paulo), Colombia (Cesar), Paraguay (Alto Paraná, Caaguazú, Caazapá, Canindeyú, Central, Cordillera, Guairá, Paraguarí), Peru (Cusco, Junín, Tumbes), and Venezuela (Falcón, Zulia).

##### Phenology.

Flowering: September to May and July; fruiting: January to December.

##### Notes.

*Tanaecium selloi* differs from other *Tanaecium* species by the foliaceous or minute and triangular prophylls of the axillary buds, and fruits without a central ridge but with margins raised. Populations from semi-deciduous and dry areas of Argentina, Southern Brazil, Bolivia, and Paraguay show leaflets that are pubescent abaxially; these features are restricted to those populations (Tab. 1).

#### 18. Tanaecium tetragonolobum

Taxon classificationPlantaeLamialesBignoniaceae

(Jacq.) L.G.Lohmann, Nuevo Cat. Fl. Vasc. Venezuela 274. 2008.

948FFE43-D29A-5376-B4FF-62BAE27C38F9

Fig. 1K, P

Bignonia tetragonoloba Jacq., Fragm. Bot. 36. 1809 [1810].
Ceratophytum tetragonolobum (Jacq.) Sprague & Sandwith, Bull. Misc. Inform. Kew 1934: 222. 1934. Type: N. J. Jacquin, Fragm. Bot. 36, tab. 40, fig. 2 1809 [1810]–illustration! (lectotype, selected by Lohmann and Taylor 2014).

##### Habitat and distribution.

*Tanaecium tetragonolobum* is found in dry to evergreen lowland forest vegetation (Gentry 1997) in Belize (Cayo, Orange Walk, Toledo), Bolivia (Beni, Chuquisaca, Cochabamba, La Paz, Pando, Santa Cruz), Brazil (Acre, Mato Grosso, Pará, Rondônia), Colombia (Atlántico, Bolívar, Chocó, La Guajira, Magdalena, Meta, Santander, Sucre), Costa Rica (Alajuela, Guanacaste, Guanaste, Puntarenas, San José), Ecuador (Napo, Pastaza), Guatemala (Petén), Guyana, Lesser Antilles (Grenada), Mexico (Campeche, Chiapas, Quintana Roo, Tabasco, Yucatán), Nicaragua (Atlántico Sur, Carazo, Chinandega, Chontales, Granada, León, Managua, Masaya, Río San Juan, Rivas), Panama (Canal Area, Darién, Herrera, Panama, Panamá, San Blas), Peru (Loreto, Madre de Dios, San Martín, Ucayali), Trinidad and Tobago, and Venezuela (Anzoátegui, Aragua, Barinas, Bolívar, Carabobo, Distrito Federal, Falcón, Guárico, Lara, Mérida, Miranda, Monagas, Portuguesa, Táchira, Yaracuy, Zulia).

##### Phenology.

Flowering: February to November; fruiting: January to December.

##### Notes.

*Tanaecium tetragonolobum* can be confused with two sympatric species, *T. jaroba* and *T. dichotomum* due to the stems with interpetiolular glandular fields (sometimes lacking in *T. dichotomum*) and subulate or bromeliad-like prophylls of the axillary buds (Tab. 1). However, *T. tetragonolobum* can be separated from *T. jaroba* by the membrane-like domatia (lacking in *T. jaroba*), lack of glandular peltate trichomes abaxially (present in *T. jaroba*), and interpetiolular patelliform trichomes < 0.3 mm (vs. interpetiolular patelliform trichomes > 0.3 mm in *T. jaroba*). On the other hand, *T. tetragonolobum* can be separated from *T. dichotomum* by the trifid tendrils (vs. simple tendrils in *T. dichotomum*) (Tab. 1).

#### 19. Tanaecium tetramerum

Taxon classificationPlantaeLamialesBignoniaceae

(A.H.Gentry) Zuntini & L.G.Lohmann, TAXON 65(5): 1059. 2016.

D436D096-ABEA-5638-AC82-63657514453C

Fig. 1I

Sphingiphila tetramera A.H.Gentry, Syst. Bot. 15: 277–279, fig. 1. 1990. Type: Paraguay. Alto Paraguay: Chovoreca, moist sandy soil along pond in open cerrado vegetation, 19°20'S 59°05'W, 12 Aug 1983, W. Hahn 1600 (holotype, MO [MO–077156]!; isotypes, G [G00094221] image!, MBM–117809 not seen, MO [MO–077155]!, NY [00328929]!, PY–3783!, US [00432848]!).

##### Habitat and distribution.

*Tanaecium tetramerum* is known from Central South America, where it occurs in Bolivia (Cochabamba, Santa Cruz), and Paraguay (Alto Paraguay, Chaco). This species occurs in xerophytic vegetation along the Chaco, in transition areas between the Chaco and Bolivian Chiquitano, Interandian, and Andean valleys. *Tanaecium tetramerum* generally grows on sandy soils or rocky outcrops.

##### Phenology.

Flowering: January to February, August and November; fruiting: January to February, April, and July.

##### Notes.

*Tanaecium tetramerum* is characterized by a series of unique morphological features that allow this species to be easily separated from other species of *Tanaecium* such as the thorn-tipped branchlets, terminal leaflets never replaced by tendrils, corollas actinomorphic, hypocrateriform, and 4-lobed (Gentry 1990; Pace et al. 2016) (Tab. 1).

#### 20. Tanaecium truncatum

Taxon classificationPlantaeLamialesBignoniaceae

(A.Samp.) L.G.Lohmann, Ann. Missouri Bot. Gard. 99(3): 467. 2014.

054F7515-E324-5441-84C6-8BA9BF8ABB07

Fig. 1J

Periarrabidaea truncata A.Samp., Bol. Mus. Nac. Rio de Janeiro 12: 86. 1936. Type: Brazil, Amazonas, Manaus, capoeira além da Villa Municipal, lugar alto, 27 July 1931, A. Ducke s.n. (holotype, RB–24093!; isotype, R–28731!).

##### Habitat and distribution.

This species occurs in humid forest vegetation in Bolivia (Pando), Brazil (Amazonas, Mato Grosso, Rondônia), and Peru (Cusco, Loreto, Madre de Dios, Ucayali).

##### Phenology.

Flowering: November to March, and May to October; fruiting: February, July to August, and October to December.

##### Notes.

This species differs from other *Tanaecium* species by the foveolate domatia, calyces basally constricted, and pale-yellow corollas (Tab. 1).

#### 21. Tanaecium xanthophyllum

Taxon classificationPlantaeLamialesBignoniaceae

(DC.) L.G.Lohmann, Ann. Missouri Bot. Gard. 99(3): 467. 2014.

458D3B23-419E-5642-B107-54504FF03926

Fig. 1K

Tabebuia xanthophylla DC., in A.DC., Prodr. 9: 214. 1845.
Arrabidaea xanthophylla (DC.) Bureau & K.Schum., Fl. Bras. 8(2): 70. 1896.
Xylophragma xanthophylla (DC.) J.F.Macbr., Publ. Field Mus. Nat. Hist., Bot. Ser., 13 (pt. 5c, no. 1): 65. 1961.
Pithecoctenium xanthophyllum (DC.) Miers, Proc. Roy. Hort. Soc. London 3: 199. 1963.
Spathicalyx xanthophylla (DC.) A.H.Gentry, Phytologia 35(3): 195. 1977. Type: Brazil, Amazonas, Alto Amazonas, Rio Negro, Maribi, towards River Japurá, Dec. 1819, C.F.P. von Martius 2967 (holotype, G-DC [G00133960]!; isotypes, M [M0088929]!, M [M0088930]!, M [M0088931]!, M [M0088932]!, M [M0088933]!, M [M0088934]!, M [M0088935]!).

##### Habitat and distribution.

This species occurs in wet forest vegetation in Bolivia (Beni, Chuquisaca, La Paz, Santa Cruz), Brazil (Acre, Amazonas, Maranhão, Mato Grosso, Pará, Rondônia), Colombia (Amazonas, Putumayo), Ecuador (Napo, Pastaza), and Peru (Amazonas, Cusco, Junín, Loreto, Madre de Dios, San Martín, Ucayali).

##### Phenology.

Flowering: October to July; fruiting: February to July and December.

##### Notes.

*Tanaecium xanthophyllum* differs from other species of *Tanaecium* by the leaflets with yellow dendritic simple trichomes, bracteoles with a proportion ≥ 4:5 to the flower pedicel, corollas with peltate trichomes in the ventral portion internally. The species epithet refers to the yellow stems, leaves, inflorescences, and fruits (Tab. 1).

### Incertae Sedis

#### Tanaecium mutabile

Taxon classificationPlantaeLamialesBignoniaceae

(Bureau & K. Schum.) L.G.Lohmann. Ann. Missouri Bot. Gard. 99(3): 465.

CD5F0C0D-09A1-52E0-B2E6-6BC052DF80D4

Arrabidaea mutabilis Bureau & K.Schum., Fl. Bras. 8(2): 38. 1896. Type: Brazil. São Paulo, Campinas [“Brésil méridional” on sheet], 16 Sep 1868, J. Correia de Méllo 44 (lectotype designated by Lohmann and Taylor 2014 P [P00468542]!; isolectotypes, P [P00468543]!, P [P00468544]!, P [P00468545]!, P [P00568546]!, S [S09-21566] image!, S as photocopy at MO–2909990!, F–999017!; F–784134!).

##### Notes.

New morphological and molecular data indicates that *T. mutabile* is nested within *Fridericia*, instead of *Tanaecium* (Frazão & Lohmann, in prep.).

## Supplementary Material

XML Treatment for Tanaecium affine

XML Treatment for Tanaecium apiculatum

XML Treatment for Tanaecium bilabiatum

XML Treatment for Tanaecium caudiculatum

XML Treatment for Tanaecium crucigerum

XML Treatment for Tanaecium cyrtanthum

XML Treatment for Tanaecium decorticans

XML Treatment for Tanaecium dichotomum

XML Treatment for Tanaecium duckei

XML Treatment for Tanaecium exitiosum

XML Treatment for Tanaecium jaroba

XML Treatment for Tanaecium kuhlmannii

XML Treatment for Tanaecium neobrasiliense

XML Treatment for Tanaecium parviflorum

XML Treatment for Tanaecium pyramidatum

XML Treatment for Tanaecium revillae

XML Treatment for Tanaecium selloi

XML Treatment for Tanaecium tetragonolobum

XML Treatment for Tanaecium tetramerum

XML Treatment for Tanaecium truncatum

XML Treatment for Tanaecium xanthophyllum

XML Treatment for Tanaecium mutabile

## Footnotes

* TAXON in press. 2019. We do not intend to publish this combination here, but in Kaehler et al. (2019, in press.)

** TAXON in press. 2019. We do not intend to publish this combination here, but in Kaehler et al. (2019, in press.)
